# Pulmonary aspergillosis in a horse: a case report

**DOI:** 10.29374/2527-2179.bjvm004723

**Published:** 2024-01-09

**Authors:** Ubiratan Pereira Melo, Cíntia Ferreira, Suziane Wilma Mota Barreto

**Affiliations:** 1 Veterinarian, DSc. Centro Universitário Mauricio de Nassau. Natal, RN, Brazil.; 2 Veterinarian. PetLab Laboratório, Natal, RN, Brazil.

**Keywords:** equine, *Aspergillus* spp., pneumonia, itraconazole, equino, Aspergillus spp., pneumonia, itraconazol

## Abstract

Respiratory diseases considerably affect equine athletes, being the second most common cause of poor performance. Among these diseases, fungal pneumonia in horses, caused specifically by *Aspergillus* spp., is relatively rare but potentially fatal. Fungal pneumonia typically affects horses exposed to fungal elements due to environmental factors, immunosuppression, or previous debilitating illnesses. Treatment is complex, with minimal success due to late diagnosis and serious concomitant underlying diseases. The choice of medication depends on the site of infection, the fungal species involved, and financial considerations. This report describes a case of pulmonary aspergillosis diagnosed in a 10-year-old castrated Quarter Horse. Transtracheal lavage revealed fungal elements characteristic of *Aspergillus fumigatus*. Treatment with dexamethasone, bromhexine hydrochloride, and itraconazole led to a successful recovery. The diagnosis of equine aspergillosis is challenging because its clinical signs overlap with other respiratory diseases. Fungal infections like aspergillosis are gaining attention in the equine health scene. Early and accurate diagnosis is crucial to avoid unnecessary use of antibiotics and prevent antimicrobial resistance. Furthermore, veterinarians and horse handlers must be aware of the risks of spreading aspergillosis to humans, emphasizing preventative measures and respiratory protection.

## Resumo

As doenças respiratórias afetam consideravelmente os equinos atletas, sendo a segunda causa mais comum de mau desempenho. Entre essas condições, a pneumonia fúngica em cavalos, causada especificamente por *Aspergillus* spp., é relativamente rara, mas potencialmente fatal. Normalmente afeta cavalos expostos a elementos fúngicos devido a fatores ambientais, imunossupressão ou doenças debilitantes. O tratamento é complexo, com sucesso mínimo devido ao diagnóstico tardio e doenças subjacentes graves concomitantes. A escolha do medicamento depende do local da infecção, das espécies fúngicas envolvidas e das considerações financeiras. Este relato descreve um caso de aspergilose pulmonar diagnosticado em equino Quarto de Milha castrado, de 10 anos de idade. A lavagem transtraqueal revelou elementos fúngicos característicos de *Aspergillus fumigatus*. O tratamento com dexametasona, cloridrato de bromexina e itraconazol levou a uma recuperação bem-sucedida. O diagnóstico da aspergilose equina é um desafio porque seus sinais clínicos se sobrepõem a outras doenças respiratórias. Infecções fúngicas como a aspergilose estão ganhando atenção no cenário da saúde equina. O diagnóstico precoce e preciso é crucial para evitar o uso de antibióticos e prevenir a resistência antimicrobiana. Além disso, os veterinários e tratadores de cavalos devem estar cientes dos riscos de propagação da aspergilose em humanos, enfatizando medidas preventivas e de proteção respiratória.

## Introduction

Respiratory diseases have a major impact on equine athletes and are often cited as the second most common cause of their poor performance. Lower respiratory tract conditions are common in adult horses, ranging from mild viral infections to serious bacterial infections (Melo et al., 2021; Melo & Ferreira, 2022).

Fungal pneumonia in horses is uncommon. The disease state tends to occur when debilitating conditions favor the penetration and growth of fungal elements. Contributory factors include exposure to overwhelming numbers of organisms in the environment, stabling of horses in moist or dusty conditions, prolonged administration of antimicrobials that disrupt the microfloral balance or interfere with vitamin synthesis, and the existence of an immunosuppressive state either primarily or secondarily due to endocrinopathy, neoplasia, or the long-term administration of immunosuppressive drugs (Higgins & Pusterla, 2006).

*Aspergillus* spp. infections cause many diseases, from localized to fatal disseminated invasive infections in humans and animals (Dobiáš et al., 2023). In animals, aspergillosis is predominantly a respiratory infection that may subsequently develop into a systemic condition (Hattab et al., 2021). Pulmonary aspergillosis, although relatively rare in horses, has been associated with enterocolitis secondary to *Salmonella* spp. infection, being less common in other enteric infections. Developed pneumonia, caused by opportunistic fungi, may be associated with a previous history of debilitating disease or an immunosuppressive condition such as colitis, peritonitis, septicemia, endotoxemia, endocrinopathy, or chronic bacterial pneumonia (Busato et al., 2020; Lima et al., 2021; Moreira et al., 2004; Trápaga et al., 2023).

The genus *Aspergillus* comprises 344 species of saprotrophic fungi, globally distributed, thermotolerant, resistant to different pHs, and isolated from various environments such as air, soil, dust, and decomposing organic matter. In the equine species, aspergillosis manifests as a condition, primarily affecting the upper respiratory tract, frequently described as impacting the nostrils, nasal cavity, and guttural pouch (Hattab et al., 2021). Immunosuppressed animals are susceptible to developing infections and, on many occasions, develop systemic aspergillosis, a clinical condition that is characteristically fatal (Lima et al., 2021).

Clinical diagnosis of aspergillosis can be challenging since its symptoms are similar to those of other respiratory diseases (e.g., retropharyngeal lymph node infection, pharyngeal lymphoid hyperplasia, pharyngeal polyps, pneumonia, and viral and bacterial infections). Clinical signs and the onset of disease vary with the causative organism; however, horses with primary fungal pneumonia often display depression, anorexia, fever, weight loss, exercise intolerance, tachypnea, nasal discharge, coughing, adventitial lung sounds, or both. Affected horses may also display symptoms related to other body systems, as some fungal pathogens can cause disseminated infection (Cafarchia et al., 2012; Higgins & Pusterla, 2006).

Although ancillary diagnostic aids (e.g., endoscopy, radiography, or ultrasonography) may be useful in suspecting infection of *Aspergillus* spp. in horses, the definitive diagnosis may require a combination of various diagnostic aids, including cytology and fungal culture of transtracheal wash (TTW) or bronchoalveolar lavage (BAL) fluid; histopathology and culture of lung biopsy; serological testing; and molecular detection of *Aspergillus* spp. from pulmonary fluid or tissue (Cafarchia et al., 2012; Higgins & Pusterla, 2006).

Treating fungal respiratory disease in horses is challenging and prevention of fungal infections is nearly impossible because of the ubiquitous nature of fungal spores in the environment. Treatment of *Aspergillus spp* pneumonia is associated with minimal success because of the rarity of early diagnosis and a concurrent severe underlying illness that is commonly present. The drug of choice depends on the site of infection, the fungus involved, and the owner's financial resources. In some cases, therapy may not be attempted because of the severity of the primary disease, expense, or poor prognosis for disease resolution (Stewart & Cuming, 2015).

This paper aims to report a case of pulmonary aspergillosis in a horse.

## Case report

A 10-year-old male, gelding, Quarter Horse used in vaquejada competitions presented with a history of cough and difficulty breathing for 1 year, which aggravated when subjected to physical exercise. On two separate occasions, equine asthma was diagnosed via clinical and endoscopic examination due to the accumulation of secretion in the trachea and changes in lung auscultation characterized by the presence of crackles and wheezing. Only mild neutrophilia was observed in the blood count on both occasions. The values obtained were 9.456 cells/mm^3^ in the first exam and 8.800 cells/mm^3^ in the second exam (reference value: 2.200-8.800 cells/mm^3^ - Lopes et al., 2021).

In both situations, the treatment comprised the use of broad-spectrum antibiotic therapy, intravenous corticosteroids (dexamethasone at a dose of 0.2mg/kg every 24 hours intravenously for 7 days), bronchodilators (Clenbuterol - 0.8µg/kg, orally every 12 hours for 2 days, followed by 3.2µg/kg administered by the same route and with the same time interval for 15 days), mucolytics (bromhexine hydrochloride - 0.3mg/kg, intravenously, every 24 hours for 8 days), and nebulization, but without remission of the clinical condition. The interval between treatments was 7 months.

According to the owner, the horse continued to present daily coughing episodes of varying intensity, exercise intolerance, and difficulty breathing at rest, even after the two therapeutic protocols. On clinical examination, the animal presented mucopurulent nasal secretion bilaterally, mixed dyspnea, and a positive cough reflex. The clinical parameters were within the reference limits for the species, except for an increase in respiratory rate (30 mrm). Lung auscultation revealed the presence of adventitious lung sounds, crepitus, and wheezing, diffusely distributed throughout the lung auscultation field in both antimeres.

Samples from the respiratory tract for cytological and microbiological analysis were obtained through transtracheal lavage. To collect the transtracheal lavage, the horse was kept in a station and after performing trichotomy and antisepsis of an area measuring 10 x 10 cm in the middle region of the neck and cranial to the bifurcation of the stenocephalic muscle, a 17-gauge cannula was introduced in the skin between the tracheal rings, reaching the lumen of the trachea. A 60.9 cm long polyethylene catheter was introduced through this cannula until it reached the carina region. About 100 mL of 0.9% sterile saline solution was introduced through the catheter, which was immediately aspirated through light suction using a syringe. The procedure was repeated until a satisfactory volume was recovered according to the methodology described by Fernandes et al. (2000).

The transtracheal fluid obtained had a cloudy appearance and blackish-colored debris. The fluid was refrigerated and immediately sent for laboratory analysis as recommended by Lessa et al. (2005).

**Figure 1:**
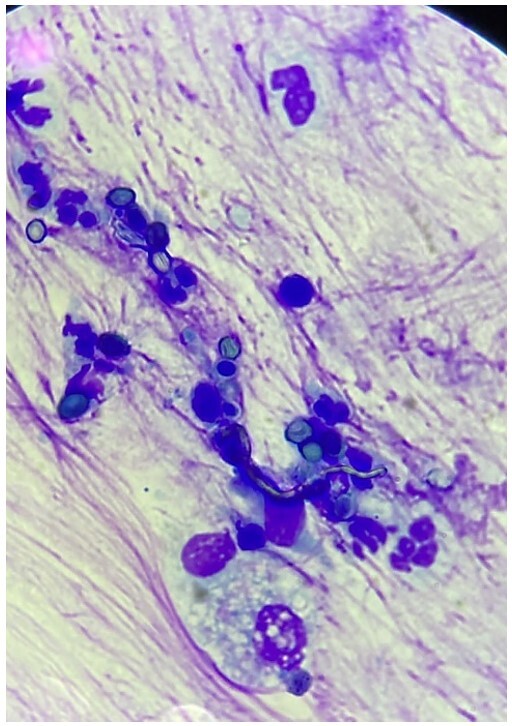
Oval structures similar to fungal conidia identified during microscopy of transtracheal lavage. The presence of macrophages is also observed on the slide. Coloration: panotic. Magnification: 40x.

Biochemical and cellular analysis of the transtracheal lavage revealed a density of 1.006, pH of 6.5, total protein value of 19 mg/dL (pyrogallol method) and 15 mg/dL (dipstick), and leukocyte count of 1,400 x10^3^/µL. Microscopy of the lavage revealed a monomorphic population of mononuclear cells, represented by foamy macrophages and reactive lymphocytes, in addition to a few degenerated neutrophils (<4%). Moreover, oval, bluish structures, similar to conidia, presenting a whitish halo, were observed in large numbers in the transtracheal lavage (Figure 1). According to the microscopic changes in the sample, the transtracheal lavage was characterized as a fungal effusion.

**Figure 2:**
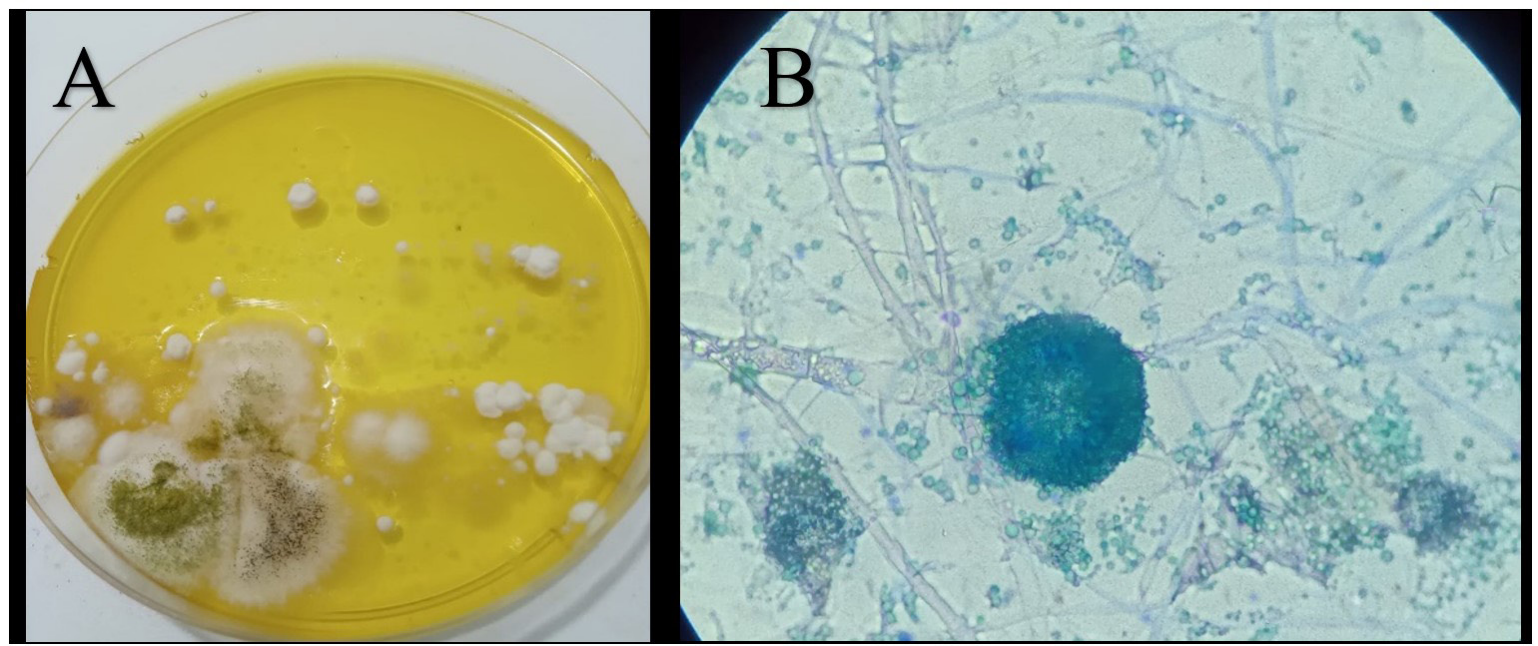
Microbiological culture of Aspergillus fumigatus. A- Colonies greenish with a granular to cottony aspect, in Sabouraud dextrose agar, after five days. B- Conidiophores with a single or bisserial aspergillary vesicle with abundant phialoconid formation.

Transtracheal lavage samples were seeded in Sabouraud Agar culture medium to isolate and identify the pathogen (Figure 2). After isolation, the agent was identified. Furthermore, under microscopy, filamentous fungi with long, branched hyphae with spherical to oval-shaped conidia at the ends of the conidiophores characteristic of *Aspergillus fumigatus* were observed.

Treatment comprised the administration of dexamethasone at a dose of 0.2mg/kg every 24 hours intravenously for 7 days (Cortvet - UCB Vet); bromhexine hydrochloride at a dose of 0.3mg/kg every 24 hours intravenously for 7 days (Aliv V- Agener União); and itraconazole at a dose of 400mg/kg every 24 hours for 45 days. As part of the treatment plan, it was recommended to maintain the animal in a semi-stabling regime, administer pelleted feed, and supply green grass. About 15 days after the initiation of treatment, the animal was re-examined, and all clinical parameters were within normal limits. On lung auscultation, no adventitious lung sounds were identified, and an evident improvement in the respiratory pattern was observed. About 45 days after the end of treatment, the animal returned to sports with no signs of recurrence.

## Discussion

Fungi are ubiquitous in the equine environment (eg, in hay, soil, and bedding), and fungal infections have been reported in horses of all ages, breeds, and occupations. Fungal respiratory disease is considered rare in horses; however, geographic variability in frequency does exist (Stewart & Cuming, 2015). Despite some reports of systemic aspergillosis in horses diagnosed at necropsy in Brazil (Busato et al., 2020; Silva et al., 2014), pulmonary aspergillosis is still rarely reported in horses (Moreira et al., 2004).

Due to the fact that spores of microorganisms of the genus *Aspergillus* are present in the environment where horses live and are natural inhabitants of the gastrointestinal flora of several animal species, the suspicion of pulmonary aspergillosis must be considered in all cases of pulmonary diseases of probable infectious origin, non-responsive to the administration of antimicrobials, cases of pulmonary disorders secondary to other infectious or allergic processes and in debilitated or immunosuppressed animals as suggested by Moreira et al. (2004).

Pathogenic fungi can be primary pathogens, capable of infecting immunologically normal horses, or opportunistic pathogens, which are capable of infecting only horses that are immunocompromised, such as those undergoing treatment with corticosteroids or with a concurrent, unrelated disease (Stewart & Cuming, 2015). No predisposing factors were identified in the present report. Despite the report of the use of corticosteroids for the treatment of a probable clinical condition of asthma, the authors believe that this was not a predisposing factor for the development of pulmonary aspergillosis, as these drugs were used in specific moments and not chronically and prolonged time.

In the present report, it is believed that infection by *Aspergillus fumigatus* occurred after inhalation of spores present in moldy hay and bedding. Stewart and Cuming (2015) emphasize that the causative organisms are able to penetrate into the distal airways and alveoli because of their small sporular diameter resulting in the manifestation of the clinical condition. There are limited reports of horses surviving pulmonary aspergillosis, comparable with human medicine, in which 50% to 90% of patients with invasive aspergillosis die despite treatment. Despite some reports in Brazil of the postmortem diagnosis of aspergillosis (Alves et al., 2018; Busato et al., 2020; Lima et al., 2021; Moreira et al., 2004; Silva et al., 2014) reported a case of pulmonary aspergillosis in a horse with a history of chronic lung disease that was successfully treated.

Corroborating the case described by Moreira et al. (2004), the initial diagnosis of the horse in this report was equine asthma, a relatively common condition in several regions of the country (Oliveira et al., 2022). In Brazil, a large country with different climatic conditions, several studies have described chronic lung conditions related to the different stages of equine asthma (Melo et al., 2007; Oliveira et al., 2022), resulting in the disease being the main suspect in cases of chronic respiratory disease in sport animals. The classic manifestation of equine asthma varies from exercise intolerance to dyspnea during rest, in addition to chronic cough, mucous to mucopurulent nasal discharge (Melo et al., 2007; Oliveira et al., 2022), findings compatible with those reported by the owner in the present report and also present in cases of fungal pneumonia (Higgins & Pusterla, 2006).

Together with the history, clinical signs, endoscopic findings, and fluid cytology, the diagnosis of asthma in horses could be considered in horses with more than 25% neutrophils (Couëtil et al., 2016). In the case reported, neutrophils corresponded to less than 4% of the cells in the tracheal lavage, excluding asthma as a possible cause of the clinical signs presented by the horse. The tracheal wash technique is well described and is widely used for diagnosing inflammation in the lower airways (Oliveira et al., 2022), and shown to have greater sensitivity and specificity for detecting increased quantities of neutrophils in airways than BAL (Rossi et al., 2018).

Clinicians must take care in attributing significance to the presence of fungal elements, free or in large quantity, in transtracheal aspirate fluid, as they are commonly identified in healthy horses or horses with severe asthma (Dobiáš et al., 2023). The fungal elements found in the transtracheal lavage should not be considered clinically relevant whenever accompanied by a normal predominance of macrophages, lymphocytes, and nondegenerate neutrophils (Stewart & Cuming, 2015), contrasting with the findings of degenerated neutrophils found in this report. Sales (2009) states that the identification of *Aspergillus* spp. in respiratory samples associated with a history of respiratory dysfunction are diagnostic criteria in pulmonary aspergillosis.

As the horse had undergone two previous endoscopies with the same findings, in the present report it was decided to perform transtracheal lavage. The fluid obtained presented several blackish-colored debris, suggestive of necrotic tissue detachment from the airways, as observed by Moreira et al. (2004).

One hypothesis for the development of pulmonary aspergillosis in the present report is the possibility that this fungal infection was facilitated by the clinical condition of asthma, as previously suspected in clinical examinations. Dobiáš et al. (2023) demonstrated that the trachea could be massively colonized by *Aspergillus* species, especially in individuals with severe asthma. Both in this report and in that of Moreira et al. (2004) it is likely that both animals presented a fungal pulmonary condition at the beginning of its evolution secondary to equine asthma. Thus, early diagnosis enabled the remission of clinical symptoms, preventing the development of systemic aspergillosis.

The treatment adopted in the present report consisted of the administration of corticosteroids and itraconazole. Although corticosteroids are related to the development of immunosuppression, in moderate to severe cases of pulmonary aspergillosis, corticosteroids improve lung function and reduce the likelihood of developing pulmonary consolidation (Sales, 2009). This same treatment protocol proved to be effective in resolving a case of pulmonary aspergillosis, as demonstrated by Moreira et al. (2004).

In recent years, the equine health scenario in Brazil has been marked by an alarming increase in the incidence of systemic or pulmonary aspergillosis in horses. Aspergillosis in horses has long been thought to be an uncommon condition in this species, but several reports indicated a paradigm shift that calls for increased awareness of the ailment. This has been corroborated by reports from numerous studies, including those by Moreira et al. (2004), Silva et al. (2014), Alves et al. (2018) and Busato et al. (2020), who documented the presence of this condition in different regions of the country.

An alarming aspect is that aspergillosis was not included in the differential diagnosis list, and the diagnosis was confirmed only through necroscopic and/or histopathological examinations. This emphasizes the need for an early and accurate diagnosis in addition to appropriate management and treatment strategies to address this emerging condition. Veterinarians and horse breeders need to be aware of the occurrence of aspergillosis in horses in Brazil and prepared to include it in their differential diagnoses, especially in cases of respiratory and systemic symptoms.

An important aspect to be considered is that horses can potentially transmit aspergillosis to humans who come into close contact with infected horses. The spores of *Aspergillus* spp. can be released to the environment through respiratory secretions and contaminated feces when a horse develops pulmonary aspergillosis or another form of the disease, putting the health of veterinarians, handlers, and other animal health professionals who work directly with these animals at risk. Proper diagnosis and treatment of affected horses, rigorous hygiene of facilities and equipment, adoption of appropriate management practices, and increased awareness among those who work with horses on the potential risks, and protective measures (for example, the use of respiratory protection masks) suggested by Medeiros et al. (2023) are important steps to stop the spread of aspergillosis to humans.

Beyond the spread of aspergillosis in humans, the consequences of delayed and incorrect diagnosis of aspergillosis can be grave, and one of the most significant concerns is the indiscriminate use of antibiotics in horses owing to inaccurate or delayed diagnoses. When aspergillosis is not identified properly and quickly, respiratory symptoms may be mistakenly attributed to bacterial infections. This can lead to the prescription of broad-spectrum antibiotics, often without medical necessity, because these medications are not effective against fungal infections and promote the development of antimicrobial resistance in bacteria present in the environment, posing a serious threat to both equine and public health, as antimicrobial resistance can make treating bacterial infections more difficult and less effective.

Therefore, the importance of early and accurate diagnosis of equine aspergillosis is not only limited to the health of horses but is also directly related to maintaining the effectiveness of antibiotics. It is imperative to increase public awareness of the risks associated with inappropriate antibiotic usage and promote evidence-based veterinary practices to address aspergillosis and antimicrobial resistance and improve equine health and public health.

## Conclusions

Diagnosing pulmonary aspergillosis in horses is challenging due to overlapping symptoms with other respiratory diseases. Although the success of treatment for pulmonary aspergillosis is limited, the early and accurate diagnosis in the present report enabled clinical remission of the symptoms. Improved awareness and diagnostic practices are needed to adequately address equine aspergillosis.
